# Evaluation of inflammatory parameters and cognitive impairment in a murine model of *Pseudomonas aeruginosa *pneumosepsis

**DOI:** 10.1186/cc13003

**Published:** 2013-11-05

**Authors:** Flora Magno, Danielle O Nascimento, Pedro CB Alexandre, Patrícia A Reis, Patrícia T Bozza, Hugo C Castro-Faria-Neto, Fernando A Bozza

**Affiliations:** 1 Immunopharmacology Laboratory, IOC/FIOCRUZ, RJ, Brazil

## Background

Sepsis is a severe medical condition characterized by systemic inflammatory response secondary to infection, which frequently progresses to multiple organ dysfunction and death. It is currently the leading cause of death in ICUs worldwide. The most frequent source of infection in sepsis is the lung with a high lethality rate. *Pseudomonas aeruginosa *is one of the most common pathogens found in sepsis patients. Cognitive impairment is a significant consequence of sepsis reported among survivors. The encephalopathy associated with systemic inflammation is not well understood so the development of new clinical relevant models to help understand this sequelae is important. In this study we aimed to evaluate acute inflammatory markers and establish a long-term consequence in a murine model of pneumosepsis.

## Materials and methods

C57/BL6 mice were submitted to intratracheal instillation of 10^5 ^colony-forming units of *P. aeruginosa*. Six hours later the bronchoalveolar lavage fluid was collected for cell migration, protein (BCA method) and cytokine (ELISA) analysis. Caudal vein blood samples were collected for cell counting. Another group of animals had their lungs perfused for myeloperoxidase quantification and histological analysis. Evan's blue dye method was used for the assessment of lung permeability. The survival rate of animals submitted to *P. aeruginosa *instillation was observed daily during 7 days. This group of animals received a single dose of antibiotic meropenem (30 mg/kg), 6 hours after pneumonia induction. Cognitive damage was evaluated through the freezing test.

## Results

Our results showed that *P. aeruginosa *infection caused an expressive recruitment of leukocytes, mainly neutrophils to the lung. Myeloperoxidase, a marker for neutrophil migration, was significantly increased in the lungs of animals instilled with *P. aeruginosa*. The animals instilled with *P. aeruginosa *also showed a significant increase in IL-6, KC and protein levels. Histological analysis showed an intense cell infiltrate in the lung tissue and the survival rate was extensively lower in *P. aeruginosa *infected mice. Additionally, the animals submitted to pneumosepsis had a loss of aversive memory 13 days after pneumonia induction and this loss remained 50 days later.

## Conclusions

Our study demonstrates the acute inflammatory response to *P. aeruginosa *lung infection and indicates that possibly this pneumonia model can cause irreversible cognitive impairment. Our results reveal a possible experimental model for the study of encephalopathy associated with systemic inflammation.

